# Gestational Weight Gain: Results from the Delta Healthy Sprouts Comparative Impact Trial

**DOI:** 10.1155/2016/5703607

**Published:** 2016-08-09

**Authors:** Jessica L. Thomson, Lisa M. Tussing-Humphreys, Melissa H. Goodman, Sarah E. Olender

**Affiliations:** ^1^United States Department of Agriculture, Agricultural Research Service, 141 Experiment Station Road, Stoneville, MS 38776, USA; ^2^Department of Medicine and Cancer Center, University of Illinois at Chicago, 1747 W. Roosevelt Road, No. 3416, Chicago, IL 60618, USA

## Abstract

*Introduction*. Delta Healthy Sprouts trial was designed to test the comparative impact of two home visiting programs on weight status, dietary intake, and health behaviors of Southern African American women and their infants. Results pertaining to the primary outcome, gestational weight gain, are reported.* Methods*. Participants (*n* = 82), enrolled early in their second trimester of pregnancy, were randomly assigned to one of two treatment arms. Gestational weight gain, measured at six monthly home visits, was calculated by subtracting measured weight at each visit from self-reported prepregnancy weight. Weight gain was classified as under, within, or exceeding the Institute of Medicine recommendations based on prepregnancy body mass index. Chi-square tests and generalized linear mixed models were used to test for significant differences in percentages of participants within recommended weight gain ranges.* Results*. Differences in percentages of participants within the gestational weight gain guidelines were not significant between treatment arms across all visits.* Conclusions*. Enhancing the gestational nutrition and physical activity components of an existing home visiting program is feasible in a high risk population of primarily low income African American women. The impact of these enhancements on appropriate gestational weight gain is questionable given the more basic living needs of such women. This trial is registered with ClinicalTrials.gov NCT01746394, registered 4 December 2012.

## 1. Introduction

Many women in the United States (US) do not gain the recommended amount of weight during pregnancy [1]. Inappropriate gestational weight gain (GWG) has been associated with gestational diabetes, gestational hypertension, preeclampsia [2–4], and operative vaginal delivery and cesarean section during birth [2, 5]. For the infant, complications associated with excessive GWG include macrosomia [1, 2], while those associated with inadequate GWG include preterm birth, being small for gestational age (SGA) birthweight, and failure to initiate breastfeeding [6]. Women who begin their pregnancy obese are at risk for the same adverse pregnancy [7, 8], delivery [9, 10], and birth complications [10] as women with inappropriate GWG. Additionally, obese women are at risk for miscarriage [11], while their infants are at risk for fetal distress, perinatal morbidity and mortality [12], still birth [13], and birth defects [14, 15]. Given these facts, the need for interventions designed to reduce inappropriate GWG is great, especially in high risk populations.

Women residing in the Lower Mississippi Delta region of the US constitute a population at high risk for adverse pregnancy, delivery, and birth outcomes as this region is characterized by high poverty, low educational attainment, and high prevalence of minorities. Among women, obesity prevalence increases as income and education decrease [16], and African American women of reproductive age (20–39 years) are more likely to be obese than their white and Hispanic counterparts (56% versus 28% and 36%, resp.) [17]. Serious concerns for this population of women and their children are raised since women who begin their pregnancy overweight/obese are more likely to gain excessive weight as compared to normal weight women [18] and African American women are more likely to have inadequate GWG as compared to non-Hispanic white women (23% versus 18%, resp.) [19].

Fortunately, a recent review of studies designed to prevent excessive GWG concluded that diet only, exercise only, or combined diet and exercise interventions can reduce the risk of excessive GWG [20]. However, few interventions have been conducted solely in populations at high risk for inappropriate GWG. To our knowledge, the Delta Healthy Sprouts trial is the first GWG diet and exercise intervention specifically designed to test the comparative impact of two maternal, infant, and early childhood home visiting (MIECHV) programs on weight status, dietary intake, and health behaviors of women and their infants residing in the rural Lower Mississippi Delta region. This paper reports results from the gestational period in terms of the primary outcome, GWG, and secondary outcomes related to delivery and infant birth characteristics.

## 2. Methods

### 2.1. Ethics, Consent, and Permissions

This study was approved by the Institutional Review Board of Delta State University (IRB protocol number 12-024). All participants gave written informed consent. Delta Healthy Sprouts trial is registered at ClinicalTrials.gov (NCT01746394).

### 2.2. Design and Recruitment

**Figure 1:**
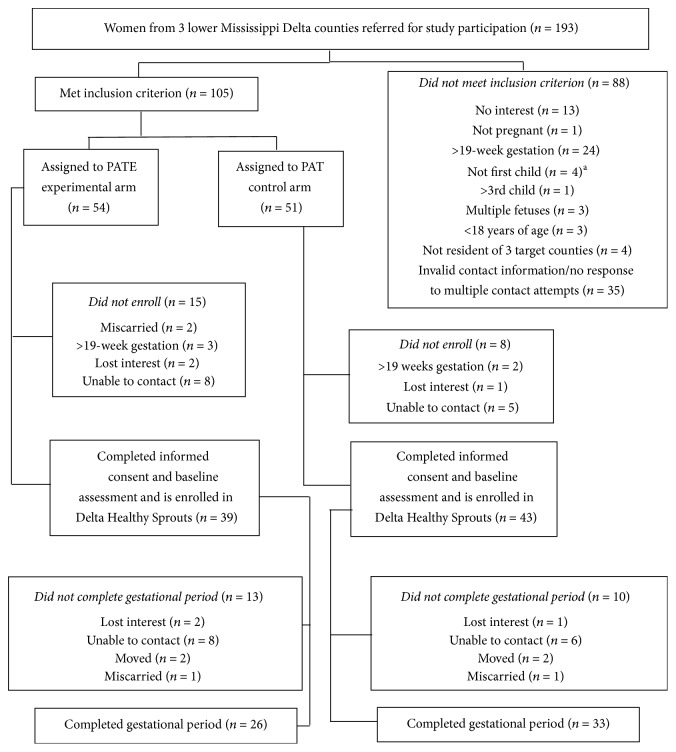
CONSORT diagram of recruitment, assignment, enrollment, and completion of gestational period for Delta Healthy Sprouts. ^a^Original exclusion criterion, later changed to pregnant with >3rd child. PAT, Parents as Teachers; PATE, Parents as Teachers Enhanced.

This study represents a longitudinal analysis of the Delta Healthy Sprouts participants' weight gain in the gestational period. A comprehensive description of the Delta Healthy Sprouts trial has been published elsewhere [21]. Briefly, 82 pregnant women participated in this trial which was conducted in three Lower Mississippi Delta counties. Inclusion criteria included female gender, at least 18 years of age, less than 19-week pregnant with first, second, or third child, singleton pregnant, and resident of Washington, Bolivar, or Humphreys County in Mississippi. Participant enrollment occurred on a rolling basis; hence baseline data were collected between March 2013 and December 2014. Data from the gestational period was collected between April 2013 and May 2015. The target enrollment was 75 women in each of the two arms of the trial (experimental and control). The sample size of 150 women was based on the following assumptions: 20% attrition rate, 37% of control participants with GWG within the Institute of Medicine (IOM) recommendations, and a 22% difference between treatment arms for GWG within recommendations. Additionally, calculations were based on a one-sided significance test, 80% power, and a type 1 error rate of 0.05 [21]. However, recruitment was stopped prior to reaching these numbers due to unexpected difficulties recruiting pregnant women meeting the study criteria. Recruitment was extended as long as possible, but fiscal concerns eventually necessitated the closing of this period.  Figure 1 illustrates the CONSORT diagram.

Delta Healthy Sprouts will evaluate the impact of the Parents as Teachers® (PAT) curriculum compared to a nutrition and physical activity enhanced PAT curriculum (PATE) on maternal GWG and postpartum weight control and childhood obesity prevention. Parents as Teachers is a nationally recognized, evidence based, home visiting program that seeks to increase parental knowledge of child development, improve parenting practices, provide early detection of developmental delays, prevent child abuse, and increase school readiness [22]. Participants were randomly assigned to one of two treatment arms [PAT control (*n* = 43) or PATE experimental (*n* = 39)] and were followed for 18 months.

### 2.3. Intervention

The control arm of the intervention was based on the PAT curriculum that included one-on-one home visits, monthly group meetings, developmental screenings, and a resource network for families. Home visitation is the key component of the PAT model where Parent Educators provide parents with research based information and activities. Materials provided were tailored to the age of the child and responsive to parental information requests.

The experimental arm of the intervention was built upon the PAT curriculum by adding culturally tailored, maternal weight management and early childhood obesity prevention components. These features were based upon foundational elements from the Diabetes Prevention Program (DPP) and the Infant Feeding Activity and Nutrition Trial (InFANT). Elements based upon the DPP principles included a flexible, culturally sensitive, individualized educational curriculum taught on a one-to-one basis [23]. Elements taken from InFANT included anticipatory guidance and parenting support principles [24]. Anticipatory guidance involves providing practical, developmentally appropriate, child health information to parents in anticipation of significant physical, emotional, and psychological milestones [25]. Parenting support emphasizes children's psychological and behavioral goals, logical and natural consequences, mutual respect, and encouragement techniques [26].

For PATE, emphasis was placed on educating mothers about healthy eating and weight control during pregnancy and the ways in which they can facilitate the development of appropriate eating, physical activity, and other health behaviors in their children, including modeling these behaviors themselves. Intervention components of the PATE arm included healthy weight gain during pregnancy and weight management after pregnancy, nutrition and physical activity in the gestational (mother) and postnatal (mother and infant) periods, breastfeeding, appropriate introduction of solid foods, and parental modeling of positive behaviors. Lessons included hands-on activities, instructional DVDs, and goal setting for both diet and exercise. At each monthly visit in the gestational period, participants were given personalized weight gain charts that contained reference ranges for the Institute of Medicine (IOM) GWG recommendations based upon prepregnancy body mass index (BMI). Participants' current as well as past weight gain from previous visits was marked on these charts.

Both arms of the intervention were delivered in the home to women beginning in their early second trimester of pregnancy by community based, trained Parent Educators. These visits occurred monthly and were approximately 60–90 minutes in length for the PAT lessons and approximately 90–120 minutes for the PATE lessons. Additional details regarding study methodology and lesson plan outlines have been published elsewhere [21].

### 2.4. Measures

Participants provided information pertaining to demographic characteristics (e.g., age, marital status, household size and income, education, employment, insurance, and prenatal care), health history and current health conditions, and delivery and infant birth outcomes (e.g., infant's birthweight and length, delivery method, problems during labor or delivery, and infant's race and gender). Premature birth was defined as less than 37-week gestation and calculated based on the difference between the self-reported due date and the infant's birthdate.

Anthropometric measures included height which was measured in duplicate using a portable stadiometer (model seca 217, Seca, Birmingham, UK) and weight which was measured using a digital scale (model SR241, SR Instruments, Tonawanda, NY). Both measures were performed without shoes or heavy clothing. Prepregnancy weight and infant birthweight were self-reported by the mothers. BMI was calculated as weight (kg) divided by height (m) squared where height was averaged if the two measures differed. Infant low birthweight was defined as less than 2,500 grams [27]. Macrosomia (high birthweight) was defined as birthweight exceeding 4,000 grams [27]. Small and large for gestational age (LGA) were defined as less than the 10th percentile and greater than the 90th percentile, respectively, based on sex specific birthweight tables for white infants and race specific birthweight tables for African American infants [28]. Race specific values were used for African American infants because percentile values were lower for both African American female and male infants at greater than 33-week gestation as compared to percentile values for white female infants. Hence, less classification errors likely occurred with the use of race specific percentiles for African American infants given all the infants in this study were at least 34-week gestation at birth.

Details regarding other measures and questionnaire data that were collected, but are not relevant to the current paper, have been published elsewhere [21]. All measures and questionnaires were collected or administered by trained Parent Educators using computer-assisted personal interviewing.

### 2.5. Statistical Analyses

Statistical analyses were performed using SAS® software, version 9.4 (SAS Institute Inc., Cary, NC). Descriptive statistics, including means, standard deviations, frequencies, and percentages, were used to summarize participants' demographic characteristics, anthropometric measures, and compliance rates for monthly visits. Compliance with visits was defined as the participant having the visit and percentages were calculated for each of the six gestational visits by treatment arm. Additionally, the total number of home visits held in the gestational period also was calculated for each participant and summarized by treatment arm.

Compliance with IOM GWG recommendations [1] was determined by first calculating participants' recommended weight gain based on prepregnancy BMI category and gestational age at each visit:

(1) Recommended  WG=rate  of  WG×gestational  age−12 weeks+2.0 kg,

where WG is weight gain; rate of WG is 0.6 kg/week for underweight, 0.5 kg/week for normal weight, 0.3 kg/week for overweight, and 0.2 kg/week for obese; 2.0 kg is upper limit for first trimester of weight gain.

Next, participants' measured weight gain was calculated by subtracting self-reported prepregnancy weight from measured weight assessed at each visit. Finally, measured weight gain was compared to recommended weight gain. Participants were classified as compliant with IOM weight gain recommendations if they fell within the lower and upper limits of the calculated (approximate) recommended weight gain based on their gestational age at that visit. For the baseline (enrollment) visit, no lower limit was used because it is not unusual for pregnant women to lose weight in the first trimester due to morning sickness [29]. For the present analyses, gestational age at each visit was calculated by inputting participants' reported due date into an online pregnancy calculator [30].

The self-reported final pregnancy weight captured at the postnatal month (PM) 1 visit was used to calculate the final GWG if it was deemed plausible. Plausibility for the self-reported final pregnancy weight was determined as follows: (1) if the last measured weight occurred at the GM 6 through GM 8 visit and the self-reported final weight was greater than this last measured weight, then the self-reported weight was defined as plausible (*n* = 7 implausible); or (2) if the last measured weight occurred at the GM 9 visit and the calculated weight gains, based on last measured and self-reported weights, were within ±10% of one another, then the final weight was defined as plausible (*n* = 3 implausible).

Because of concerns with possible misreporting of prepregnancy weight and the substantial proportion (51%) of participants who exceeded IOM recommendations for GWG at the enrollment visit [31], an additional method of classifying GWG was utilized. The rate of weight gain between the GM 4 (enrollment) and last GM visits (or plausible self-reported final pregnancy weight as outlined previously) was computed. Participants' rates were then classified as under, within, or exceeding IOM recommended weight gain rates based on prepregnancy BMI category. Using this method allowed for detection of a potential intervention effect irrespective of GWG that occurred prior to study enrollment and underreporting of prepregnancy weight.

Chi-square tests of association or Fisher's exact tests (categorical measures) and two sample *t*-tests (continuous measures) were used to assess differences between PAT and PATE participants' baseline characteristics and GWG rate and between gestational period study completers' and noncompleters' baseline characteristics. Study completers were defined as participants who had their GM 9 visit or those who had at least two visits in the gestational period and their PM 1 visit. The second definition was used because a substantial proportion of PAT and PATE participants (36% and 42%) who had their PM 1 visit missed their GM 9 visit due to the early birth of their infant. Generalized linear mixed models were used to test for significant differences in proportions of participants under, within, or exceeding IOM GWG recommendations across all gestational period visits. However, because of convergence issues, the outcome was dichotomized to within or not within (versus under, within, or exceeding) IOM recommendations. The model employed a logit link function, random intercept for subject, variance component covariance structure, and Laplace approximation for the maximum likelihood estimation. Although maximum likelihood estimation is an approach for handling missing data in repeated measures, a sensitivity analysis also was conducted in which the last measured weight (or plausible self-reported final gestational weight) was used as the final weight and compared to IOM GWG recommendations. A chi-square test of association was used to assess differences between the two treatment arms.

## 3. Results

Gestational period retention rates for the PAT and PATE treatment arms were 77% (33/43) and 67% (26/39), respectively.  Table 1 presents comparisons between treatment arms for baseline characteristics. The majority of both PAT and PATE participants were African American (95% and 97%), were single (91% and 95%), received Medicaid (93% and 90%), were overweight/obese prior to pregnancy (63% and 72%), and started their prenatal care in their second month of pregnancy (56% for both). Additionally, PAT and PATE participants were young (mean age = 23 ± 4.6 years and 23 ± 4.7 years) and early in their second trimester of pregnancy (mean gestational age = 17 ± 1.9 weeks and 18 ± 2.4 weeks). There were no significant differences between PAT and PATE participants at baseline. However, when comparing study completers to noncompleters, significantly more study completers owned or had access to a motor vehicle as compared to noncompleters at baseline (95% versus 78%, *P* = 0.036; data not shown in Table 1).

### 3.1. Compliance Rates and Pregnancy Characteristics

**Figure 2:**
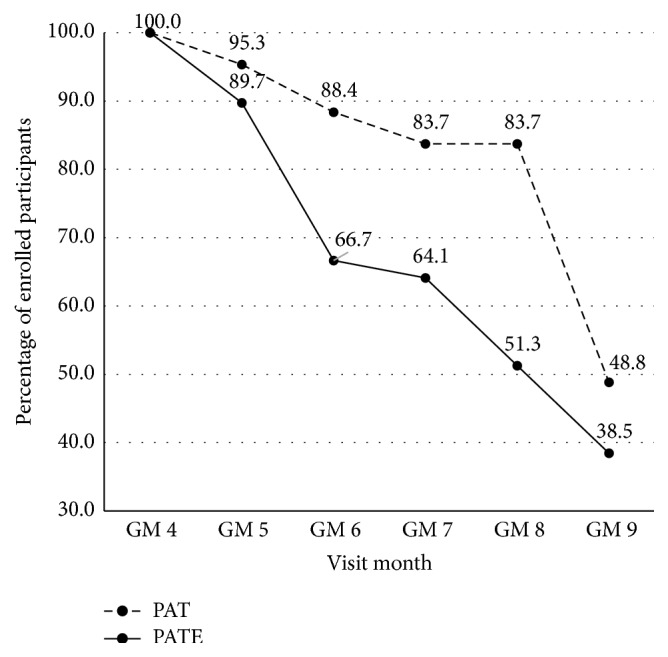
Delta Healthy Sprouts participant compliance rates in the gestational period by treatment arm.

Figure 2 and Table 2 present compliance rates for participants by treatment arm. As seen in Figure 2, after enrollment (GM 4 visit) compliance rates were lower for PATE as compared to PAT participants for GM 5 through GM 9 visits. Results presented in Table 2 indicate that over three-fourths (79%) of PAT participants had at least 5 of the 6 monthly visits as compared to approximately half (51%) of PATE participants.

Table 3 presents comparisons between treatment arms for pregnancy characteristics and outcomes. The majority of PAT and PATE participants received advice from their prenatal care provider concerning healthy eating (88% and 83%) and exercise (81% and 80%). However, significantly more PAT participants received advice concerning weight gain (55%) as compared to PATE participants (29%). Additionally, more PAT participants reported receiving recommended weight gain amounts that were concordant with IOM guidelines (67%) as compared to PATE participants (33%), although this difference did not reach statistical significance.

### 3.2. Gestational Weight Gain

Table 4 presents results of the GWG analysis categorized by treatment group, GM visit, and IOM recommendation class (under, within, and exceeding). Although the results are presented by three IOM classifications, for modeling purposes, under and exceeding IOM GWG recommendations were collapsed into a single class. Based on the generalized linear mixed model, differences in percentages of participants within the IOM guidelines for GWG were not significant between treatment arms across all GM visits. Likewise, differences in percentages of participants within the IOM guidelines for GWG were not significant between treatment arms in the sensitivity analysis (using last measured weight). Additionally, the difference in percentages of participants within the IOM guidelines for rate of GWG (between GM 4 and last GM visit) was not significant between PAT and PATE treatment arms (15% versus 12%, Fisher's exact test *P* = 1.0; data not shown in Table 4).

### 3.3. Delivery Outcomes and Infant Birth Characteristics

Table 5 presents delivery outcomes and infant birth characteristics by treatment arm. Approximately one-fourth of the participants (23% of PAT and 25% of PATE) delivered their infants by C-section. Additionally, 17% of PAT and 8% of PATE infants were born premature and 7% and 21%, respectively, were SGA, while 7% and 13%, respectively, were LGA. Only the percentages of female infants (30% in PAT and 58% in PATE) differed significantly between treatment arms.

## 4. Discussion

Results from the gestational period of the Delta Healthy Sprouts trial indicated that, contrary to our hypothesis, the enhanced version of the MIECHV program did not have a larger positive impact on GWG as compared to the standard version of the program. Further, the results do not support the hypothesis of improved delivery and infant birth outcomes (i.e., reduced complications) for PATE experimental participants as compared to PAT control participants.

Others also have reported a lack of intervention effect on GWG in terms of IOM recommendations. In a Swedish study, the proportions of pregnant women who exceeded IOM recommendations for GWG did not differ between the low cost intervention (recommended GWG, weight gain monitoring, and prescribed exercise) and standard maternity care groups [32]. Likewise, no intervention effect was found for weight gained during pregnancy or excessive GWG in a two-armed health coaching and education study conducted with pregnant Australian women [33]. In contrast, a significantly lower proportion of German women receiving lifestyle counseling (recommended GWG, weight gain monitoring, and diet and exercise goal setting and monitoring) exceeded IOM recommendations as compared to the standard prenatal care control group [34]. However, significantly more overweight/obese women were in the control group (31%) as compared to the intervention group (16%) which may have affected the intervention's efficacy given the fact that overweight/obese women are more likely to gain weight excessively during pregnancy as compared to healthy weight women [18]. Additionally, only 21% of the participants in the German study were overweight/obese before becoming pregnant as compared to 67% in the current study. It may be that achieving GWG within IOM recommendations via lifestyle intervention is harder with overweight/obese women than healthy weight women as suggested in a recent review of diet, exercise, and combined diet and exercise GWG interventions [20]. Supporting this supposition, a statistically significant and positive intervention effect for adherence to IOM GWG recommendations was found in prepregnancy healthy weight Latina women but not in prepregnancy overweight or obese women participating in an active lifestyle pregnancy intervention [35]. Given these results, further research is needed to determine if interventions designed to optimize GWG need to be specifically tailored for prepregnancy BMI status.

Similar to our study, significant differences in delivery and infant birth outcomes were not found between intervention and control groups in the German pregnancy lifestyle counseling study [34] or between the health coaching and education alone groups in the Australian study [33]. Likewise, no differences in adverse birth outcomes were found in the active lifestyle pregnancy intervention for Latina women [35] or the Swedish study [32], although both studies indicated they were not powered to detect differences between treatment groups. Small sample size also was an issue for the current study given that recruitment was stopped prior to achieving the planned numbers of participants and the small number of adverse delivery and birth outcomes reported.

Several unanticipated factors may have adversely affected the PATE treatment's impact. The lower prevalence of reported prenatal care provider advice about GWG coupled with the higher rate of discordant reported advice (as compared to IOM recommendations) observed in the PATE as compared to the PAT arm is concerning. In a prospective cohort study of primarily African American pregnant women, those who reported receiving advice that was discordant with IOM recommendations were over three times as likely to gain excessive GWG as compared to women who reported receiving concordant advice [36]. Further, results from a qualitative study, conducted with overweight/obese women after the birth of their first child, suggest that women value the opinion of their prenatal care provider more than other sources of information (i.e., books, magazines, friends, family, and the Internet) [37]. Taken together, this may help explain why there was no apparent impact of the PATE treatment on achieving GWG within IOM recommendations.

It is not clear why less PATE participants received advice about GWG from their prenatal care provider. It is possible that more PATE participants received care from a provider who did not routinely (or correctly) advise his/her patients about IOM recommendations for GWG. However, this hypothesis cannot be confirmed as we did not collect information about the participants' prenatal care provider.

Another possible reason for lack of PATE impact involves the relatively high percentage of participants (49% for PAT and 54% for PATE) who exceeded IOM recommendations for GWG at the initiation of the study (i.e., prior to intervention). It is likely that we intervened with these women too late in their pregnancy to have a significant impact on their subsequent GWG. Indeed, visual inspection of GWG graphs suggests that the majority of participants in this study remained within the IOM recommendation classification (i.e., under, within, or exceeding recommendations) in which they began the study. Future studies are needed to determine the most optimal time to intervene during a woman's pregnancy in order to achieve the most positive effect on GWG.

The lower compliance rates for the PATE participants as compared to the PAT participants were unexpected. However, the lower compliance in the PATE arm was greatly influenced by the majority (85%) of noncompleters withdrawing early from the study (after the first or second visit) as compared to 40% of the PAT noncompleters. Analysis of participant satisfaction surveys completed in the gestational period indicated that the intervention was well received in both treatment arms (PATE and PAT mean scores = 4.6 and 4.5 points, resp.; maximum score = 5). However, the longer length of the PATE visits (approximately 90 minutes) as compared to the PAT visits (approximately 60 minutes) coupled with discussions on eating healthy and obtaining adequate physical activity may have adversely affected the retention rate of the PATE participants. Residents of the region targeted in this study are predominantly African American with high rates of poverty, child poverty, births to unmarried women, and low birthweight infants [21]. Hence, discussions concerning diet and exercise may have been somewhat under- or overwhelming for participants given their more basic needs, such as stable housing and/or safe living conditions, food security, dependable transportation, long term/full time employment with benefits, educational attainment, enduring phone service, and affordable and reliable child care. It may be that long term improvement in the nutritional status of impoverished individuals requires policy efforts to reduce or eliminate socioeconomic disadvantage along with nutrition intervention [38].

The higher proportions of SGA and LGA infants in the PATE arm as compared to the PAT arm, although not statistically significant, were not as hypothesized and concerning. We checked for possible associations with GWG rate and pattern, prepregnancy BMI, prenatal care provider advice about GWG, gestational diabetes and hypertension, and maternal stature [39]. Interestingly, all seven of the SGA infants were born to participants whose rate of GWG exceeded IOM recommendations. Similarly, all five of the LGA infants were born to participants whose overall pattern of GWG exceeded IOM recommendations. While we could find no studies in the literature that confirmed or refuted our SGA finding, the LGA finding does confirm results previously reported in the literature [40, 41]. However, further investigation of GWG for participants with SGA infants revealed some unusual patterns. Rates slowed considerably in the second or third trimester for three (one PAT and two PATE) participants which supports findings reported by others [42]. Additionally, premature SGA infants were born to two (one PAT and one PATE) participants with gestational hypertension, also supporting associations reported by others [43]. For the remaining two (PATE) participants with SGA infants, one had both an excessive pattern and rate of GWG, while the other gained no weight until GM 7. So while this participant's GWG rate was excessive between GM 7 and GM 9, overall she gained less than the IOM recommended amount, thus supporting previously reported associations between SGA and inadequate GWG [6]. Future analyses will explore potential associations with secondary outcomes, such as dietary intake, physical activity, and psychosocial measures (e.g., depression and stress).

This study had several strengths including intervention in a population at increased risk for inappropriate GWG and adverse pregnancy and birth outcomes [21], education to dispel common pregnancy misconceptions (e.g., recommended GWG, safety of the fetus during exercise) [44], and behavior change techniques (e.g., behavioral self-monitoring) combined with dietary and physical activity goal setting [45]. Additionally, the multiple methods used to analyze GWG provided a more comprehensive view of this outcome. Limitations of this study included the self-reported prepregnancy weight which may have led to misclassification of prepregnancy BMI and over- or underestimation of GWG. However, recent evidence suggests high concordance between self-reported weight and weight measured at the first prenatal visit for prepregnancy BMI classification [46]. Further, we attempted to mitigate this potential misreporting by computing the rate of weight gain only during the gestational intervention period (i.e., between GM 4 and GM 9). While the 77% retention rate in the PAT arm was similar to the 79% reported by the PAT Director of Research & Quality Improvement (Dr. Guskin, personal communication, 2014), it was substantially lower (67%) in the PATE arm. Despite this, overestimation of the benefit of the PATE curriculum is unlikely since no differences between treatment arms were found. Finally, stopping recruitment prior to reaching the target enrollment number of 150 women limited our ability to detect significant differences between the two treatment arms. Difficulties in recruitment involved nonactive referral by prenatal care providers and Special Supplement Nutrition Program for Women, Infants, and Children local health department nutritionists, moving of one clinic to a smaller site that could not accommodate onsite recruitment by research staff, lack of a dedicated staff member for study recruitment, and competition for this population of pregnant women by other programs operating in the same area (e.g., Delta Health Partners Healthy Start Initiative) [47].

## 5. Conclusion

Enhancing the gestational nutrition and physical activity components of an existing MIECHV program is feasible in a high risk population of Southern, primarily low income, African American women. However, the impact of these enhancements on appropriate GWG is questionable given the more basic living needs of such women. Two of the greatest challenges are intervening early in pregnancy and ensuring sufficient intervention dose via home visits in the gestational period.

## Figures and Tables

**Table 1:** Delta Healthy Sprouts participant baseline (early second trimester of pregnancy) demographic characteristics by treatment.

Characteristic	PAT (*n* = 43)		PATE (*n* = 39)		*P*
	*n*	%	*n*	%	

Race					1.000
African American	41	95.3	38	97.4	
White	2	4.7	1	2.6	
Marital status					0.678
Single^a^	39	90.7	37	94.9	
Married	4	9.3	2	5.1	
Education level					0.684
9th–11th grade	7	16.3	7	17.9	
High school/GED	15	34.9	12	30.8	
Some college/technical	17	39.5	13	33.3	
College degree	4	9.3	7	17.9	
Employment status					0.468
Full time/part time	13	30.2	16	41.0	
Unemployed (looking)	21	48.8	14	35.9	
Homemaker/student	9	20.9	9	23.1	
Household monthly income^b^					0.284
<$500	10	23.3	5	12.8	
$500–$1,000	10	23.3	9	23.1	
$1,001–$1,500	4	9.3	12	30.8	
$1,501–$2,000	7	16.3	5	12.8	
$2,001–$4,000	6	14.0	3	7.7	
Do not know/refused	6	14.0	5	12.8	
Smoker in household	12	27.9	12	30.8	0.776
Smoker^c^					0.112
Current	3	7.0	1	2.6	
Stopped before pregnancy	1	2.3	0	0.0	
Stopped after becoming pregnant	2	4.7	0	0.0	
No	37	86.0	38	97.4	
Medicaid health insurance	40	93.0	35	89.7	0.703
Receiving SNAP	35	81.4	27	69.2	0.200
Receiving WIC	38	88.4	31	79.5	0.271
Own/access to vehicle	39	90.7	35	89.7	1.000
Receiving prenatal care	43	100.0	39	100.0	
Start of prenatal care (month)^d^					0.255
First	11	25.6	6	15.4	
Second	24	55.8	22	56.4	
Third–fifth	8	18.6	11	28.2	
Gestational diabetes	0	0.0	1	2.6	0.476
Gestational hypertension	2	4.7	3	7.7	0.665
Prepregnancy weight class^e^					0.386
Underweight (BMI < 18.5)	4	9.3	3	7.7	
Healthy weight (18.5 ≤ BMI < 25)	12	27.9	8	20.5	
Overweight (25 ≤ BMI < 30)	9	20.9	10	25.6	
Obese (BMI ≥ 30)	18	41.9	18	46.2	

	Mean	SD	Mean	SD	*P*

Age (years)	23.3	4.58	22.7	4.69	0.537
Household size	3.8	1.62	4.1	1.78	0.406
Gestational age^f^	17.4	1.85	17.7	2.43	0.533

PAT, Parents as Teachers control treatment; PATE, Parents as Teachers Enhanced experimental treatment; SNAP, Supplemental Nutrition Assistance Program; WIC, SNAP for Women, Infants, and Children; BMI, body mass index.

^a^Includes 1 participant who indicated she is divorced.

^b^Comparison: ≤$500–$1,500 versus $1,501–$4,000; do not know/refused excluded.

^c^Comparison: no versus all other responses.

^d^Comparison: first month versus second through fifth month.

^e^Based on self-reported prepregnancy weight; comparison: underweight or healthy

weight versus overweight or obese.

^f^Based on reported due date; enrollment data collected late for 3 participants.

**Table 2:** Delta Healthy Sprouts participant compliance with home visits in the gestational period by treatment.

Total number of home visits	PAT (*n* = 43)		PATE (*n* = 39)		*P* ^a^
	*n*	%	*n*	%	
1	1	2.3	4	10.3	0.018
2	3	7.0	6	15.4	
3	1	2.3	3	7.7	
4	4	9.3	6	15.4	
5	15	34.9	9	23.1	
6	19	44.2	11	28.2	

PAT, Parents as Teachers control treatment; PATE, Parents as Parents as Teachers Enhanced experimental treatment.

^a^ *P* value for dichotomized compliance (1–3 versus 4–6 visits).

**Table 3:** Delta Healthy Sprouts participant pregnancy characteristics^a^ by treatment.

Characteristic	PAT (*n* = 42)		PATE (*n* = 35)		*P*
	*n*	%	*n*	%	
Gestational health conditions^b^					
Hypertension	3	7.1	1	2.9	0.399
Preeclampsia	1	2.4	0	0.0	0.358
Advice by prenatal care provider					
Healthy eating	37	88.1	29	82.9	0.513
Exercise	34	81.0	28	80.0	0.916
Weight gain (WG)	23	54.8	10	28.6	0.021
WG advice by visit					NA
GM 5	12	52.2	6	60.0	
GM 6	6	26.1	1	10.0	
GM 7	1	4.3	3	30.0	
GM 8	4	17.4	0	0.0	
WG advice by BMI class^c^					NA
Underweight (BMI < 18.5)	2	8.7	0	0.0	
Healthy weight (18.5 ≤ BMI < 25)	8	34.8	2	20.0	
Overweight (25 ≤ BMI < 30)	6	26.1	3	30.0	
Obese (BMI ≥ 30)	7	30.4	5	50.0	
Amount of WG advised^d^					0.163
Under IOM recommendations	2	13.3	0	0.0	
Within IOM recommendations	10	66.7	2	33.3	
Exceeded IOM recommendations	3	20.0	4	66.7	

PAT, Parents as Teachers control treatment; PATE, Parents as Teachers Enhanced experimental treatment; WG, weight gain; NA, not applicable (counts too small to make meaningful comparisons); BMI, body mass index; IOM, Institute of Medicine.

^a^Information collected during gestational months 5 through 9 visit.

^b^Questions asked at each gestational visit; frequencies represent cumulative count for gestational months 5 through 9. Those reporting condition at enrollment are excluded.

^c^Based upon self-reported prepregnancy weight.

^d^Not all participants who reported being advised provided an amount.

Comparison: within versus under or exceeding.

**Table 4:** Delta Healthy Sprouts participant IOM recommendation classification for gestational weight gain by visit and treatment.

Visit month	PAT (*n* = 43)						PATE (*n* = 39)						*P* ^a^
	Under		Within		Exceeded		Under		Within		Exceeded		
	*n*	%	*n*	%	*n*	%	*n*	%	*n*	%	*n*	%	
4	15	34.9	7	16.3	21	48.8	13	33.3	5	12.8	21	53.8	0.079
5	10	24.4	4	9.8	27	65.9	13	37.1	3	8.6	19	54.3	
6	9	23.7	7	18.4	22	57.9	8	30.8	1	3.8	17	65.4	
7	8	22.2	8	22.2	20	55.6	8	33.3	2	8.3	14	58.3	
8	6	16.7	9	25.0	21	58.3	5	25.0	2	10.0	13	65.0	
9	5	23.8	7	33.3	9	42.9	6	40.0	2	13.3	7	46.7	
SR	4	17.4	7	30.4	12	52.2	5	27.8	1	5.6	13	72.2	
LMW	9	20.9	11	25.6	23	53.5	11	28.2	4	10.3	24	61.5	0.192

IOM, Institute of Medicine; PAT, Parents as Teachers control treatment; PATE, Parents as Teachers Enhanced experimental treatment; SR, self-reported final pregnancy weight; LMW, last measured weight.

^a^First *P* value corresponds to generalized linear mixed model using all values (except LMW) with dichotomized outcome (within or not within recommendations); second *P* value corresponds to chi-square test of association using only LMW value.

**Table 5:** Delta Healthy Sprouts participant delivery outcomes and infant birth characteristics by treatment.

Characteristic	PAT (*n* = 30)		PATE (*n* = 24)		*P*
	*n*	%	*n*	%	
Delivery mode^a^					0.887
Vaginal (not induced)	18	60.0	13	54.2	
Vaginal (induced)	5	16.7	5	20.8	
C-section (planned)	4	13.3	2	8.3	
C-section (unplanned)	3	10.0	4	16.7	
Birth complications^b^	2	6.7	2	8.7	1.000
Infant gender: female	9	30.0	14	58.3	0.036
Infant ethnicity: non-Hispanic	29	96.7	24	100.0	1.000
Infant race					1.000
African American	29	96.7	23	95.8	
White	1	3.3	1	4.2	
Premature (<37 weeks gestation)^c^	5	16.7	2	8.3	0.443
Low birth weight (<2500 g at birth)	2	6.7	5	20.8	0.221
Macrosomia (>4000 g at birth)^d^	2	6.7	2	8.3	1.000
SGA (<10th percentile)^e^	2	6.7	5	20.8	0.221
LGA (>90th percentile)^e^	2	6.7	3	12.5	0.646

PAT, Parents as Teachers control treatment; PATE, Parents as Teachers Enhanced experimental treatment; SGA, small for gestational age; LGA, large for gestational age.

^a^Comparison: vaginal versus C-section.

^b^Other than premature delivery; complications: low blood sugar, sickle cell anemia, and jaundice. 1 PATE participant response missing.

^c^Based on conception date (calculated using online pregnancy calculator and self-reported due date).

^d^No infant exceeded 4500 g at birth; 4 of 5 LGA infants are also classified as having macrosomia.

^e^Based on sex specific birthweight percentiles for 2 white infants and race specific birthweight percentiles for 52 African American infants.
